# The burden of care, quality of life and depression in relatives of patients with serious mental illness treated at Lentegeur Hospital

**DOI:** 10.4102/sajpsychiatry.v24i0.1312

**Published:** 2018-09-27

**Authors:** Sihle Nhlabathi, John Parker, Bonginkosi Chiliza, Lebogang Phahladira

**Affiliations:** 1 Department of Psychiatry, Stellenbosch University, South Africa; 2 Department of Psychiatry and Mental Health, University of Cape Town, South Africa; 3 Department of Psychiatry, Nelson R Mandela School of Medicine, University of KwaZulu-Natal, South Africa

## Abstract

**Background:**

The association between caregiver burden, quality of life and depression in patients with mental illness in a resource-limited setting is underresearched. Factors associated with caregiver burden may be amenable to intervention.

**Aim:**

To describe the level of caregiver burden and its association with quality of life and depression.

**Methods:**

The cross-sectional study was carried out in a psychiatric hospital in the Cape Flats, which is an urban area on the periphery of Cape Town. Data were collected from 104 caregivers of patients attending the outpatients service. Caregiver burden was measured using the Zarit Burden Interview questionnaire. The PHQ-9 questionnaire was to screen and measure severity of symptoms of depression. The WHOQOL-BREF was used to measure the subjective evaluation of the quality of life.

**Results:**

Most caregivers were female (76.92%), and caregivers were mild to moderately stressed (mean ZBS score 33.38 ± 21.59) and experienced moderate depression (mean PHQ-9 score). These effects were significantly associated with quality of life in psychological and social domains.

**Conclusion:**

Caring for patients with mental illness can lead to immense physical and psychological distress, leading to poor quality of life. Strategies that may reduce the burden of care may include improving the patients’ quality of life and addressing psychosocial support, and clinicians should consider screening for symptoms of depression in caregivers.

